# Precarious livelihoods at the intersection of fishing and sand mining in Cambodia

**DOI:** 10.1007/s13280-023-01963-9

**Published:** 2023-12-09

**Authors:** Furqan Asif, Lukas Van Arragon

**Affiliations:** 1 Environmental Policy Group, Wageningen University, Hollandseweg 1, 6706 KN Wageningen, The Netherlands; 2 Centre for Blue Governance, Aalborg University, Rendsburggade 14, 9000 Aalborg, Denmark; 3 School of International Development and Global Studies, University of Ottawa, 120 University Private, Room 8005, Ottawa, K1N 6N5 Canada

**Keywords:** Cambodia, Fishing livelihoods, Precarious livelihoods, Sand mining

## Abstract

Fishing and sand mining in Cambodia may not appear to have much in common. However, digging deeper reveals important parallels. Both fishing and sand mining support livelihoods and are connected to a limited natural resource. Meanwhile, they are both typified by precarious livelihoods, on the one hand, and overexploitation, on the other. In bringing these two topics together, the paper combines empirical qualitative research from two separate studies conducted by the co-authors in Cambodia, one in coastal fishing villages and another in the sand mining industry along the Mekong River. We argue that the interplay between fishing and sand mining has paradoxical impacts on livelihoods, supporting one group while undermining another. Using a precarity analysis lens, we show how an unconventional, and largely invisible frontier of natural resource exploitation—sand mining—is intertwined with fisheries, and expands our understanding of the relationship between precarious labour, environmental change, and livelihoods.

## Introduction

For millions of people around the world, particularly those in the ‘Global South’, precariousness is, and has been, a constant everyday condition (Munck 2013). Precarity is a broad concept that encompasses the overall state of insecurity and instability in people’s lives, including economic, social, and pyschological dimensions. Precarity as a concept has also expanded beyond being about people and into the non-human world: we have precarious urbanization (Souza and Samora 2022), precarious energy infrastructure (Smith-Nonini 2022), precarious political institutions (Lund 2001), and precarious cultural heritage (e.g. artefacts, monuments). While the debate around the concept and term is outside the scope of this paper (see Millar 2017 for a comprehensive overview), here we focus on a particular type of precarity: labour precarity (also termed ‘precarious work’ and ‘precarious labour’—we use these terms interchangeably). At its most basic, precarious work is understood as work that is *uncertain*, *unstable*, and *insecure* from the perspective of the worker (Hewison and Kalleberg 2013). A key driver of increasing labour precarity across all these countries is ‘flexibilization’, a term used to refer to a trend towards maximizing “flexibility” for employers via use of short-term contracts, casualization, labour supply agencies, and migrant workers, both domestic and foreign (Arnold and Bongiovi 2013).

Precarious forms of employment (e.g. work that is atypical, irregular or nonstandard, temporary or seasonal work, part-time work, and contingent employment) also shifts risks associated with such work to the workers, thereby reducing risks for businesses (i.e. employers) and/or governments. Formally, precarious employment is defined as “work for renumeration characterized by uncertainty, low income, and limited social benefits and statutory entitlements” (Vosko 2011, p. 2). The value in using the lens of precarity as a labour condition is that it goes beyond the traditional, binary understanding of “formal” (or standard) and “informal” (or nonstandard) work as these cannot fully capture the contemporary modalities of employment. An example of this is the rise of the so-called ‘gig economy’ (e.g. ride-hailing and food delivery services) which touts itself as a (formal) job creation engine, yet the same time, their ‘employees’ are in actuality subcontractors with little protections or security—a feature that is a hallmark of precarious work. As Munck (2013) observes, “there is not…a dichotomy between the formal and informal economies but rather a continuum based on considerable synergies and grey overlapping areas” (p. 755). Overall, labour precarity refers to specific conditions of labour in the modern, globalized configuration of capitalism (Munck 2013).

Precarious work has been analysed in a variety of contexts in the Global South such as migration of villagers from the farm resulting in precarious non-farm employment and a new form of poverty in India (Breman 2007) and rural precarity in farming households in Nepal (Rigg et al. 2016). Within Southeast Asia, precarious work has been analysed in Indonesia, Thailand, the Philippines, and Vietnam (Hewison and Kalleberg 2013). However, precarity analysis has tended to focus on specific sectors (e.g. informal) (Ofreneo 2013) or modalities within precarious work (e.g. labour outsourcing) (Tjandraningsih 2013) but not as much on work at the intersection of different sectors. In this paper, we focus our attention on the crossroads of two natural resource-based livelihoods in Cambodia: fishing and sand mining.

During peak fishing season (August–December), the fisheries sector (freshwater and marine combined) employs six million people (full-time, part-time, and seasonal) (Un et al. 2015; Estepa et al. 2016).^1^ In other words, fishing involves a significant proportion of labour in the country. Coastal Cambodia is a region where the ties between fishing, labour, and livelihoods are most prominent. Coastal provinces have some of the highest density of fishers in the country (Nasielski et al. 2013) and 78% of coastal households surveyed sold seafood for income (compared to 50% for those in the Tonle Sap region) (NIS 2016). Meanwhile, fishery resources have collectively been eroding. For example, recent analysis of the lower Mekong basin fishery using fish catch data from a 17-year time period shows fish catch has declined by 87.7% (Chevalier et al. 2023). Likewise, coastal fishing communities in Cambodia have been feeling the gradual decline in fisheries resources since the 1990s (Bann 1997). Such a degradation in fisheries resources has made fishing into an increasingly precarious livelihood (Kusakabe and Sereyvath 2019).

From the mid-2000s, an emerging challenge for coastal communities has been sand mining activity occurring along the coast and within the mangrove estuaries, which has had widespread ecological impacts and exacerbated the progressive decline in catch, according to fishers (Marschke 2014; Asif 2019). At the same time, the Mekong River has also been facing declines in catch, partly due to hydropower dams in the wider basin which have affected fish migrations, river hydrology, and sediment transfers (Soukhaphon et al. 2021), and exacerbated by sand mining activities.^2^ At this confluence is where the story of two natural resource extraction activities—fishing and sand mining—intersect (UNSD 2020).

The ‘sand boom’ in Cambodia has created an industry—and a demand for labour—driven by demands for river/marine sand by countries such as Singapore (John and Jamieson 2020). Like fishing, the sand industry in Cambodia is characterized by precarious employment conditions, including work in remote and isolated locations (e.g. on barges on the Mekong River or the sea), separation of family members when labourers leave their household and hometown for work in the industry, and unpredictable cycles of intermittent work. Like fishers who often must sell to middlepersons who then go on to sell to markets for a relatively larger profit, most of the wealth from sand mining does not accrue to the labourers doing the activity itself. For fishers, there are few alternative livelihood opportunities in and around the village that can earn them a living wage. For sand labourers, sand mining often offers a livelihood opportunity for them when they have few other livelihood opportunities in their home provinces. These dynamics present in both fishers and sand labourers livelihoods typify precarity, particularly the kind where the employee takes on all risks associated with the job (instead of employers or the government) and gets little social protection and statutory entitlements (Kalleberg and Hewison 2013).

Our paper aims to explain how the drive to extract sand from the Mekong River and (historically) coastal areas of Cambodia is interwoven with fisheries and reveals the interplay between precarious labour, resource extraction, and livelihoods. To do so, we adopt and apply a precarity lens as a descriptive tool as a means of analysing fishing and sand mining, following the approach of Marschke et al. (2020) who use precarity vis-à-vis livelihoods to understand the intersection of fishing and ecological change in coastal Jamaica. In doing so, we aim to reveal the parallels and paradoxes that encapsulate the novel intersection of these two resource frontiers in Cambodia.

The paper is divided as follows. In the following section, we provide a brief overview of the historical evolution of natural resource exploitation in Cambodia. Next, we describe the methods used and the empirical data collected by each co-author that forms the basis of this paper. From there, we describe the concept of labour precarity and operationalize it in the context of sand mining and fishing in Cambodia as a means of revealing insights the activities. This is followed by a section that aims to provide rich descriptions and context of livelihoods, first covering coastal fishing livelihoods and then sand mining livelihoods. We then move into a discussion that analyses the livelihoods of fishers and sand labourers through a precarity lens to show similarities, differences, and seeming contradictions between the two forms of livelihoods. Finally, we provide concluding remarks on the utility of adopting the lens of precarity to understand the intersection of interrelated natural resource-based livelihoods and invite other researchers and scholars to look at similar contexts elsewhere in the Global South.

### The quest for natural resources in Cambodia: Forest, land, fish, and (now) sand

Systematic exploitation of the forests began in the 1880s (Le Billon 1999).^3^ By the 1980s, widespread logging ramped up as demand from neigbouring Thailand and Vietnam increased, and as the forest was no longer protected as a side effect of earlier conflicts and trade embargoes by the west (Le Billon 2000).^4^ By the late 1990s, a complex web of patronage networks had emerged, in which well-connected individuals were granted exclusive rights to forest resources, further enabling the forests’ exploitation (Le Billon 2000).

This model of politically connected extractivism evolved into large-scale, illicit land acquisitions of the Cambodian countryside, known as Economic Land Concessions (ELCs) (Schoenberger et al. 2017; The Land Matrix 2021). ELCs allowed companies to clear-cut forests for agriculture, thereby depriving local people of their livelihoods. As of 2013, 700 000 farmers had been displaced from their land as a result of ELCs expanse (Neef et al. 2013).

Like land and forests, abundant fishing resources are an important part of the natural resource-based livelihood fabric of Cambodian society. However, a lack of regulation and management throughout Cambodia’s history has been a persistent challenge to fisheries’ sustainability. Despite efforts from the 2000s onward to improve fisheries management, conflicts over fisheries emerged, especially in the Tonle Sap region (FACT and EJF 2001). The conflicts followed a similar pattern to exploitation of forests and land: politically well-connected individuals were granted licenses by the Cambodian state to large fishing lots, under an agenda of “fisheries privatization”, leading to the destruction of small-scale fishers’ livelihoods (Sneddon 2007). This system of politically connected extractivism has more recently also supported exploitation of another resource, albeit one that has received less attention: sand.

Sand mining in Cambodia emerged in the 1990s and expanded rapidly thereafter (Bravard et al. 2013). In the early 2000s, sand mining in Southeast Asia gained attention for its damaging impacts on river ecosystems and erosion. This led to poorly enforced bans on inland sand mining in the late 2000s, and calls for sand export bans (Vong 2009; Narim and Paviour 2016; Thompson 2017). Only in 2017 were sand exports ‘officially’ banned (Lamb et al. 2019) in response to United Nations trade data which showed that Cambodia exported 72 million tonnes of sand to Singapore from 2007 to 2017 (Thul 2017). Although exports did in fact drop dramatically after 2017 (UNSD 2020), sand mining in Cambodia continued. Cambodia has developed its own needs and markets for river sand, a resource necessary to sustain Cambodia’s growing cities. Phnom Penh, Cambodia’s capital and its most populated city, is situated in a low floodplain and surrounded by large wetlands. Driven by the interests of foreign investors and Cambodia’s elite, sand is required to fill in the wetlands to prepare land suitable for building luxury ‘satellite cities’ (Guest 2016). Cambodian government data published in the Southeast Asia Globe magazine in 2020 show that about 9 million cubic metres (around 17 million metric tonnes) of sand was dredged in Cambodia in 2019. Experts argue, however, that the actual annual amount of sand dredged could be anywhere from three to five times the official figure (McCready 2020). An alliance of Cambodian NGOs estimated in a July 2020 report that 100 million tonnes of sand would be required to fill in a swamp area to make way for a large satellite city (Knaus 2020).

While sand is no longer exported in great quantities, the Cambodian sand industry is still global in nature. Foreign capital investment supports the urbanization boom and land reclamation in the country. The planned development, ‘ING city’, for example is funded in large part by foreign firms (Phnom Penh Post 2019). Overall, sand is simply among the more recent natural resources that have historically been exploited by an array of actors, most connected and embedded within the neopatrimonial state (Kimchoeun et al. 2007).

## Materials and methods

**Fig. 1:**
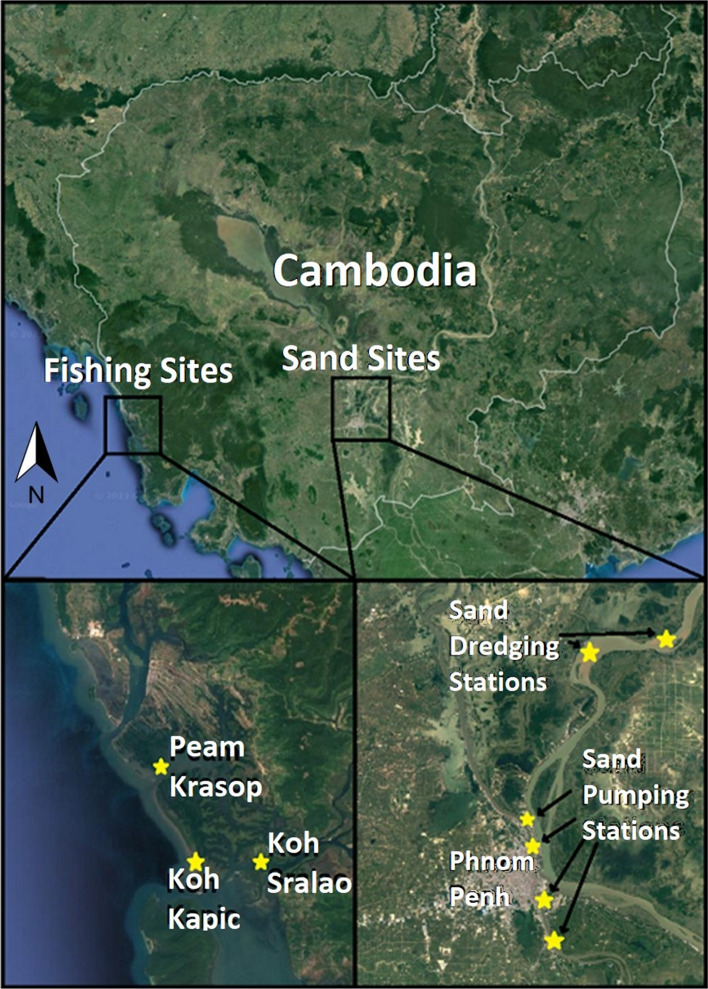
Map of study sites: fishing sites in Koh Kong and sand mining sites along the Mekong River. *Source* Google satellite imagery, modified by authors

Using qualitative data gathered from three coastal fishing villages in Koh Kong and two villages in Kampong Cham along the Mekong River, and various sand transportation sites along the Mekong in and around Phnom Penh (Fig. 1), we bring empirical insights into the extant literature that juxtaposes the lives and livelihoods of coastal villagers and fishers with those of sand mining labourers. Our study uses desktop research of secondary data on extraction industries in Cambodia to complement analysis of primary data collected through our respective fieldwork activities. Empirical data used in this paper come from two separate studies. Data related to coastal fisheries and associated livelihood dynamics (e.g. impact of sand mining, out-migration, etc.) are drawn from the first author’s doctoral field research, conducted over 18 months (2015–2017) in southwest Cambodia (Koh Kong). The fieldwork spanned three villages (Peam Krasaop, Koh Sralao, and Koh Kapic) and two urban areas (Koh Kong town and Phnom Penh) and involved a combination of 69 informal and 115 formal interviews and a mix of unstructured, semi-structured, and structured interviews (Table 1). The data were analysed using qualitative analysis software (NVivo) and followed inductive and deductive coding (see Asif 2020).

**Table 1:** Number of participants across five locations: three fishing villages in Koh Kong and two urban areas, with select demographic factors. *Source* Asif (2020). *Note* Age data were not obtained for all Phnom Penh participants (< 25%) and were thus excluded. Average age is of combined genders (i.e. not gender disaggregated)

Location	Interviews (informal)	Interviews (formal)	Scoping survey	Males	Females	Average age	Average # of years in village
Peam Krasaop	16	28	16	35	13	43	15
Koh Sralao	22	33	22	47	15	48	20
Koh Kapic	31	22	31	43	10	49	29
Koh Kong town	–	13	N/A	2	11	23	–
Phnom Penh	–	19	N/A	11	5	–	–
Total	69	115	69	138	54	–	–

Meanwhile, data on the sand mining industry are drawn from fieldwork done by the second author, collected over 2 months (October–November 2019) in Cambodia. Respondents included those working in the sand industry along the Mekong River. Field sites consisted of six sand pumping stations along the Mekong River in the capital region, two dredging stations in the Mekong River in Kampong Cham province, one sand barge travelling between Phnom Penh, and the dredging stations in Kampong Cham. Open-ended interviews were conducted with ten sand pumpers, three sand dredgers, two boat captains, and one business person who sold sand. Overall, a total of 29 interviews with 40 participants were conducted (some of these were group interviews) (Table 2). The interviews were semi-structured and covered various topics regarding the sand mining industry and working within it, such as the challenges labourers face on the job, and why they chose to enter the sand mining industry (for further details, Qualitative data were analysed through the pattern coding method, see van Arragon (2021).

**Table 2:** Interviews related to sand mining research by interviewee type and various laborer’s in sand mining. *Source* van Arragon (2021)

Type of interviewee	# Interviews (# of participants in brackets)
Sand Businesspeople	1 (1 participant)
Civil Society (NGO) Workers	4 (4 participants)
People affected by the sand extraction industry (total)	7 (17 participants)
Affected by infill (living in wetland near Phnom Penh)	1 (1)
Affected by dredging (living along the Mekong River)	6 (16)
Labourers in sand mining (total)	17 (18 participants)
Pumpers (emptying barges or filling in wetland)	5 (6)
Pumping Station Managers	4 (4)
Dredgers (operating machinery to extract river sand)	2 (3)
Khmer Boat Captains	3 (3)
Vietnamese Boat Captains	2 (2)
Total	29 (40 participants)

### Conceptual framework

Our study aims to illuminate the precarious nature of both fishers’ and sand miners’ livelihoods, employing the concept of labour precarity. Rodgers (1989) proposed four dimensions to precarious work: (i) temporal precarity, or the uncertainty that work will continue; (ii) organizational precarity, or the lack of control over wages, location, etc.; (iii) protectional precarity, or the lack of protection from injuries or other work hazards; and (iv) economic precarity, or the lack of sufficient pay to keep workers out of poverty. It is important to note that Rodgers (1989) as well as other prominent labour precarity scholars (cf. Standing 2016) conceptualize precarity through a narrow, Westernized lens, viewing precarious labour as the opposite of traditional ‘Fordist’ employment in which workers hold long, stable, and satisfying jobs (i.e. such as those found in Ford Motor Company factories).

Cambodia, however, has a vastly different labour context. Unlike Western countries, Cambodia’s labour market has not followed the traditional Fordist model. One important distinction is that most of the income-generating activities that people engage in are part of a wider informal economy, which operates outside of traditional institutional structures. As such, most (if not all) of those working within the informal economy are devoid of social protections, rendering them highly vulnerable to shocks and stressors. Moreover, most Cambodians—including both sand labourers and fishers—continue to rely on natural resources for their livelihoods. In this context, labour precarity cannot be analysed without an understanding of the environmental context, and of the implications of environmental change. Until recently, precarious labour has not been applied in fisheries work or looked at how ecological conditions influence poor working conditions. Scholars such as Marschke et al. (2020) directly address the role of unsustainable natural resource extraction in labour precarity in the developing world. In their study of coastal fishers in Jamaica, Marschke et al. (2020) show that as fish stocks are exhausted near the shoreline, fishers must travel further and further out to sea, thereby increasing the risks of boat accidents and increasing the cost of fuel and repairs. In explaining this in the context of precarious work, they also expand on the traditional concept of precarious labour developed by Rodgers (1989) by adding two more components: temporal and spatial effects and ecological and biophysical change. We adopt this revised understanding of precarious analysis to understand fishers and sand labourers’ livelihoods. Indeed, these two additional components are centrally important to fishers in Cambodia, whose livelihoods depend on natural resources and timing of livelihood activities such as fishing according to seasonal variations and whose livelihood challenges partly stem from environmental change.

Environmental factors also cause a different type of movement for work: migration. With the destruction of one’s natural resource-based livelihoods from environmental degradation, many rural Cambodians decide to leave their homes and migrate in search of work (Asif 2019, 2020). Migration can also be thought of as a form of spatial precarity, as Cruz-Del Rosario and Rigg (2019) argue how people who must migrate for work often experience precarious conditions not just in their jobs, but in their entire way of life. For example, migrant workers often leave behind their families and children, leading to emotional burdens and risks associated with living in a place without a community. This can create precarious living conditions both for the people travelling for work, as well as those left behind (Casas-Cortés 2014; Bernards 2019).

By examining the livelihoods at the intersection of fishing and sand mining through the lens of precarity, we reveal the relationship between two seemingly different kinds of work that are rarely considered together.

## Results

### Coastal fisheries and livelihoods

**Fig. 2:**
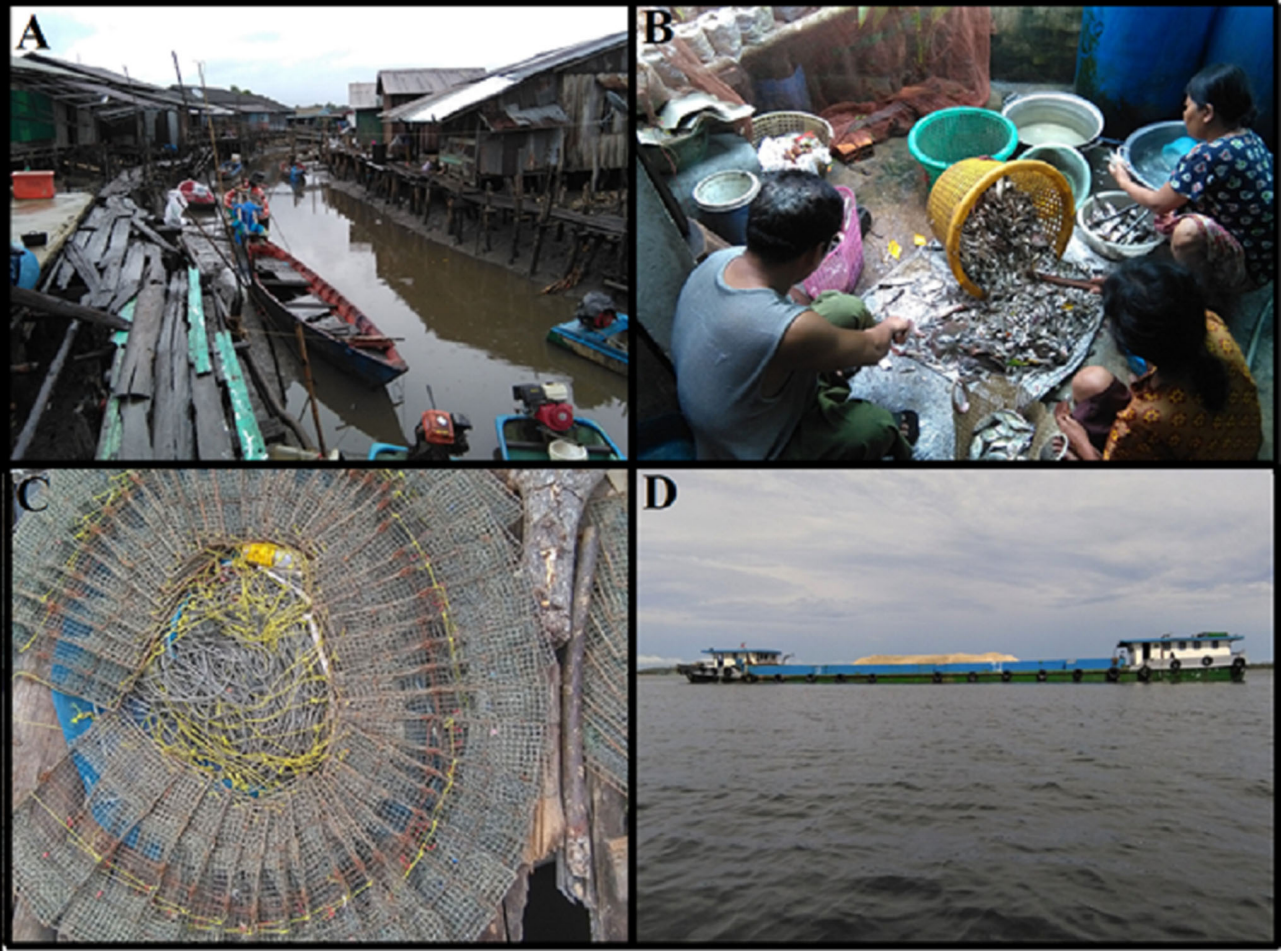
**A** A view looking onto an estuary channel of a coastal fishing village showing the typical types of boats used by villagers. **B** A family in a coastal village sorting their catch. **C** A fisher lays out his crabs traps in preparation for their next trip. **D** A barge carrying sand mined somewhere around Koh Kong province heading back to the mainland. Photos by Furqan Asif

Households in coastal fishing villages live in houses made of wood covered by a tin roof, perched on long poles above the water (Fig. 2A). This location not only keeps their home and belongings safe from tidal and storm surges but also provides easy access to their boats and the sea, the means with which they provide for their livelihood. Fishers typically go out in the early hours around dawn, fish for a few hours (e.g. setting nets and traps), returning for a break midday and then going out again. On average, they spend 8–10 hours at sea fishing (depending on the type of fishing), typically five to six days in a week but some fishers go every day. The type of fishing depends on the relative socio-economic level of households (Marschke 2012). Wealthy households set large traps 5–6 m below the sea surface or use a large circular net while less wealthy households fish using gill nets or crab traps (Fig. 2C). Once they harvest their catch, family members come together to help sort/process the catch and organize fishing gear for the next trip (Fig. 2B). In general, the division of labour in the fishing village is gendered where typically males (e.g. fathers and sons) go out to do the fishing and females (e.g. mothers and daughters) help sort and process the catch and, for example, peel crabs (the peeled crab is sold to local markets and also goes across the border to Thailand).

Coastal small-scale fishing is largely an ‘entrepreneurial’ activity and fishers run their operation independently from one another. Most fishers also typically own the means of ‘production’, i.e. catching fish, and buy all of the things needed such as a boat, an engine, and fishing gear. Fishers in coastal villages (and elsewhere in Cambodia, including its freshwater fisheries) also choose how long and often they work, within the parameters of the environment and the seasons. In this way, small-scale fishers have a high amount of autonomy in the pursuit of their livelihoods. However, this autonomy is juxtaposed by the fishers being compelled to work five or six days per week because they are catching significantly less than in the past. As one female villager explained, “fishing is decreasing, we catch less and less. Like every day we earn 10 000 KHR [~ $2.50 US]”.

Alongside having to spend more time at sea and catching less, fishing as a livelihood is filled with uncertainty and risk. On a typical day, a fisher may ask themselves questions such as ‘How much fish will I catch?’, ‘Will my fishing gear get stolen?’, or ‘What if I get sick and am unable to go fishing?’. When they are out at sea fishing, and something goes wrong, for instance, engine trouble or weather starts to turn hazardous, they face the risk of being stranded or injured. Moreover, fishers and their families have to cope with the fact that fishing is a costly livelihood. For example, new fishing gear must be bought every couple of years, at best (e.g. the seawater corrodes metal frame of crab traps easily), netting must be replaced (or fixed), boat engine parts may need replacing (or, in some cases, a new engine bought altogether), bait must be bought (when it is not caught), and gasoline needs to be stocked. Fishers are also restricted by the seasonality of fishing which is typically done during the ‘dry season’ (November–April). During the ‘rainy season’, most fishers cannot go fishing because of the potential for storms and high waves, which make conditions at sea dangerous. Altogether, with the combination of decreasing catch, and hence, decreasing incomes, alongside fixed (or, in some cases, increasing) expenses, most households have taken on substantial debt burdens (supplied by the now-ubiquitous microfinance services). Considering the declining sustainability of marine resources, exacerbated by the ecologically destructive practice of sand mining (Fig. 2D), and the uncertainty and risk that comes with being a fisher, it becomes clear that fishing as a livelihood has become increasingly precarious. One villager in Koh Sralao village who is part of a multi-generational fishing family noted in an interview that he has not intentionally encouraged any of his children to go into fishing because of its unpredictability (i.e. sometimes you catch a good amount, others you do not) and precariousness as a livelihood. Echoing these sentiments of some days being better than others, the village chief from Koh Kapic village says, “it is a risky venture, some days you earn, some days you do not earn, and it depends on the season, if it is a good season or not”.

While most of the older generation has stuck to fishing despite these challenges (and some likely because of being entrapped by debt burdens), the younger generation has viewed the possibility of fishing as their future livelihood with trepidation after seeing their parents in such precarity. Indeed, one of the most common responses by coastal fishing households has been out-migration of one (or more) household members, typically the son(s) or daughter(s). During a group interview of factory workers, one young woman explained, “I moved to Koh Kong city due to my [family’s] livelihood being poor, so I needed to find a job to earn more income to support my family”, which was echoed by another villager-turned-migrant-worker, who noted, “me too, I have to earn more money to help my family. My family could not earn much income as before and they get sick very often, so could not rely on their incomes anymore”. These choices involve a variety of trade-offs. For example, trading the security and feeling of community of the village with the insecurity of the city, as one migrant woman in Phnom Penh explained:

It [migration] affects my relationship with my family because they are in Koh Sralao, my parents worry about me more than when I was staying with them in Koh Sralao. They are calling to check how I am. Recently, it has not been safe, because a lot of robberies have occurred in my area. (Asif 2020, p. 155).

For many households, the remittances their migrant family members send back form an essential component to meeting basic needs and covering expenses. However, these measures are likely temporary, and do nothing to change the factors contributing to the increasing precarity of the central livelihood of coastal villages: sand mining, and more broadly, environmental change. Even if sand mining in the coastal areas truly ceased, coastal villagers would still have to contend with impacts from sand mining in nearby rivers and recent hydropower construction along the Tatai River. These activities have been documented to significantly alter the natural flow of sand from rivers and reduction of sediment supply, which cause erosion of the land–sea interface (Kastl et al. 2013). Koh Kapic, for example, is located downstream of Tatai River and has been dealing with increasing estuary infilling from increased sedimentation upstream. Alongside low water levels, these impacts have led to sedimentation within the estuary channels (IUCN 2013), thereby making navigating fishers’ boats difficult if not impossible, particularly during low tide or dry season (the primary time they need to go out and fish). In addition, sand mining activities that occur in areas where fishers set up their traps and gear often results in their gear being damaged or destroyed, resulting in a considerable negative, acute impact on their ability to earn a living (first author’s doctoral thesis reference). Sand mining activity also has a direct and immediate negative impact on coastal ecosystems, for example, through the destruction of seagrass beds, which act as key habitats for marine species, some in their juvenile life stage, and others (e.g. crab) as more permanent habitats (Reasey 2014). The activity also damages the mangrove estuary area, with some cases where the sand mining equipment rips out mangrove trees, further degrading the ecosystem (Marschke 2014). As one fisher from Koh Kapic fishing village relates in *The Cambodia Daily*, “the big ships pump out sand and cause the mangrove trees to fall down, killing off the vital fish and crab habitat the community depends on” (Reaksmey 2015). Moreover, sand mining has also affected the distribution of fish and crab populations by impacting the migration routes of marine organisms due to the increasing turbidity in areas where sand mining occurs (Marschke 2014). Given that fishers often rely on their accumulated local ecological knowledge (e.g. where fish and crab tend to be) to know where and when to fish, these changes result in increasing fishing effort (i.e. more time and money vis-à-vis increased petrol use) and impact overall earnings from fishing. Overall, a multitude of direct and indirect drivers have caused fishing as a livelihood for coastal fishers to become increasingly untenable and making situations for households so dire that out-migration of family members remains for some a last-ditch effort to counter the precarity faced.

### Sand mining and livelihoods

The Cambodian sand industry is carried out by private companies, which dredge sand from the Mekong River, transport it in barges, and offload and move the sand to various infill and construction sites. According to the Ministry of Mines and Energy, as of December 2020, 95 companies were officially licensed to extract sand in Cambodia (Haffner 2020). One labourer said that the company he worked for operated around 40 riverbank offloading stations. If each station had six to ten pumpers, which the labourer said was typical, the company would have employed 240 to 400 pumpers. With 83 companies actively extracting and transporting sand, there are likely thousands of sand labourers in Cambodia.

**Fig. 3:**
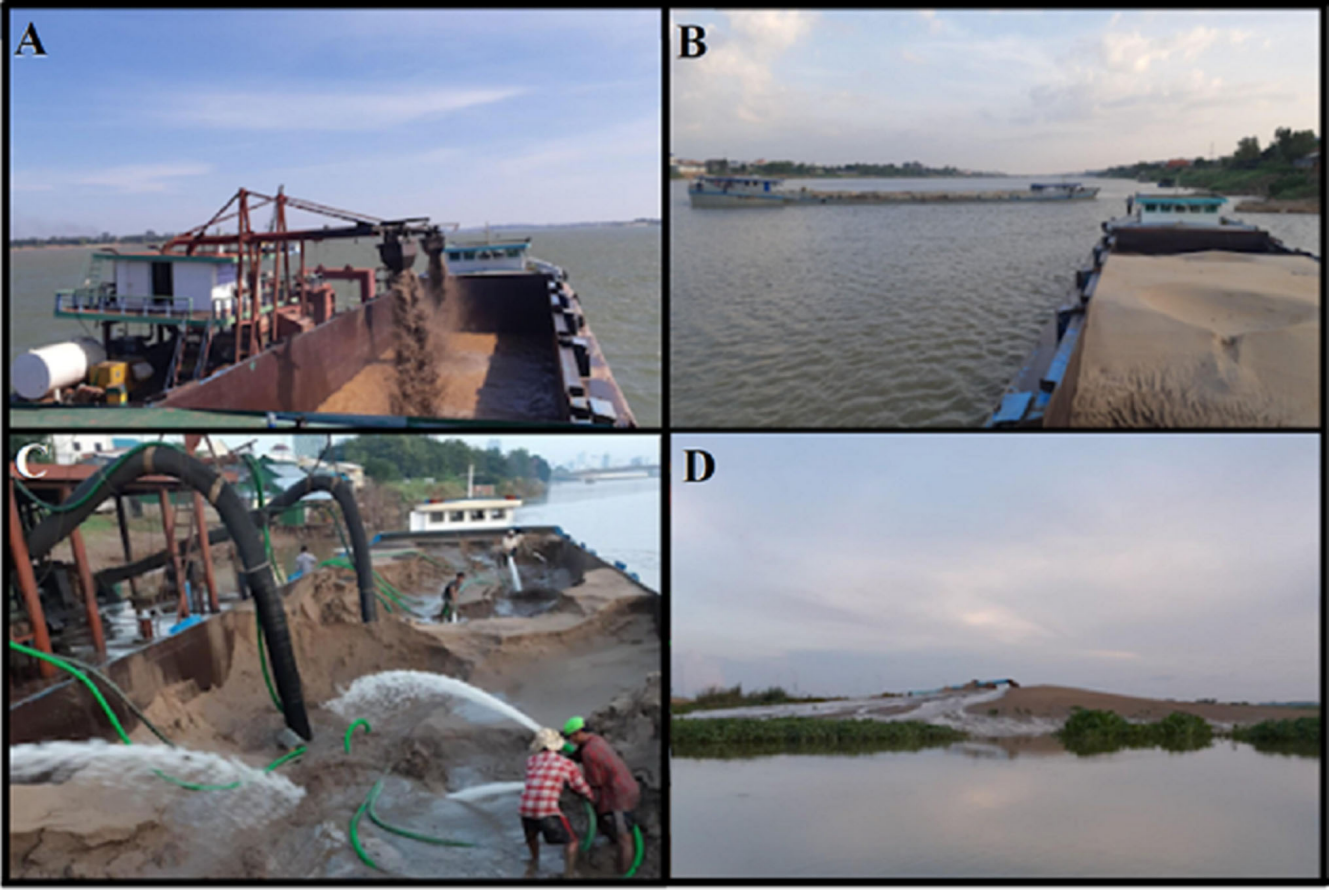
**A** Dredging sand in Kampong Cham province. **B** Transporting sand by barge to Phnom Penh. **C** Pumpers liquefying sand so it can be pumped onto land. **D** Wetland south of Phnom Penh filled with sand to prepare land for real estate development. Photos by Lukas Van Arragon

The sand commodity chain starts with dredging stations, where two labourers per station operate dredging machinery to load barges with sand (Fig. 3A). Once a barge is filled, a captain and one assistant transport the sand to a location along the river where the sand is needed. When the barge reaches an offloading station along the riverbank, a team of six to ten labourers use hoses to liquefy the sand so that it can be pumped onto land through a large engine powered tube (Fig. 3C). A “station manager”, who oversees hiring and firing labourers as well as operating the engine of the pump, leads each offloading station. After the sand is pumped off the barge, it is either deposited in a storage site to be transported to construction sites by trucks, or it is pumped directly into a wetland infill site through long rubber tubes. In the case of sand being pumped for kilometers on land through tubes, two or three pumps are required along the span of the tube (each operated by one or two people) to maintain pressure in the tube. Up to 16 people are involved in the extraction and transportation of one load of sand (Fig. 3).

Sand labour is seen as an attractive option for rural Cambodians seeking employment opportunities. For barge captains, sand work pays well. For pumpers, station managers, and dredgers, sand work is attractive because it is open to anyone who is willing to work, not requiring special skills to carry out. Furthermore, dredging and pumping work is less physically demanding than equivalent jobs in the construction sector. As one worker explained:

Sand dredging is better than construction because the pay is about the same, but in construction you do heavy labour eight or more hours a day. Here it’s a lot less busy. (Interview, November 26, 2019).

Despite these benefits, however, sand labourers’ livelihoods remain precarious. Rather than being able to enhance their livelihoods, workers make difficult livelihood trade-offs. To understand these trade-offs, it is helpful to examine the previous livelihood context of those who enter the sand industry. Most people enter the sand industry from a place of vulnerability, poverty, limited educational opportunities, and few livelihood options. Farmers sometimes face droughts or land grabbing, making it more difficult to carry out farming, while fishers often face conditions of overfishing and climate change reducing their catch (Asif 2019). Despite this context of vulnerability, Cambodians who decide to leave their home provinces still have something to lose. Rural Cambodians often possess deep knowledge and skills related to their rural occupations, whether fishing, farming, or another occupation. Rural Cambodians often live in tight knit communities that help each other in times of need by lending equipment or cash. Rural Cambodians also often possess a range of assets that can help them earn a livelihood, such as land, equipment, and free access to natural capital. A typical rural Cambodian household may earn a living through various means, such as working the land, selling wares, and gathering food from the wild to supplement their diets or incomes.

Rural Cambodians who migrate for sand work must compromise on many of these assets. Sand pumping and dredging are considered low skill jobs and do not offer workers a way to utilize or build upon their previously developed skills. Migrant sand workers from rural provinces must also leave behind physical and natural capital they may have used to pursue rural livelihood strategies. Sand workers use their company’s equipment, but do not own any of it. The lack of autonomy over resources means that in contrast to fishers for example, who are sometimes able to fish longer and further out at sea to compensate for declining fish resources, sand labourers have few ways to compensate for periods in which sand companies offer them little work. The lack of natural and physical assets also leaves workers with fewer ways to diversify their sources of income and food. Home villages therefore offer a base of livelihood and food security that sand work does not. In fact, because of the COVID-19 pandemic, many Cambodians working in cities had to return home to live off the land once more (World Bank 2021).

Where many labourers may have benefited from kin networks, group membership, and a voice in their local communities where they grew up, sand work far from their hometowns forces labourers to leave behind their social networks. This means that sand labourers are less able to rely on kin and community to provide financial or other support during difficult times. All but one of the sand labourers interviewed lived away from their families, only seeing them every few months or less. Instead, sand labourers must develop networks and relationships with their colleagues with whom they live, eat, and sleep.

## Discussion

### Fishing and sand mining: precarious livelihoods at the intersection of two resource frontiers in Cambodia

On the surface, fishing and sand mining in Cambodia may not appear to have a lot in common. However, digging deeper reveals key parallels within the livelihoods of people engaging in both activities. The most fundamental is that both are tied to natural resources that are collectively in decline, although in the case of fishing there is a possibility for it to be sustainable. Another commonality is that both are part the so-called “informal economy” that provides little social protection measures, although this is by no means unique to fishing and sand mining but also applies to other livelihoods not only within Cambodia but also across countries in the Global South (Carr and Chen 2002). The difference between the fishing and sand mining livelihoods is that the driver of precarity is different. For fishing, it is the decrease in marine natural resources that is primarily driving the precarity, with part of this decline attributed to sand mining (Marschke 2014; Thompson 2017; Lazaro 2019). For sand mining, the precarity stems from the actual labour conditions surrounding the extraction of the natural resource (Marschke et al. 2021). A second similarity is that both are connected to water with the livelihoods intimately tied to this element. Fishers live above the water and spend large parts of their days on it for fishing while sand labourers spend weeks at a time on a sand barge on the water. During this time, both fishers and sand labourers are away from their family and have to contend with changing conditions (e.g. weather). Migration is a key component for both fishers and sand mining labourers and the driver is similar, namely, decreasing viable livelihood options at home (Cruz-Del Rosario and Rigg 2019).

Analysing the livelihoods of fishers and sand labourers through a precarity lens reveals important intersections between these two seemingly disparate kinds of work (Table 3). Sand mining and fishing are both fairly labour demanding activities, requiring working with equipment and lifting and loading, etc., and neither follow a typical eight-hour work schedule. Moreover, there is no formal institutional affiliation or representation for fishers and sand labourers in Cambodia. From an organizational viewpoint, this means that both groups have no unions to represent their rights, no work contracts, and no formal recourse for garnished or missing wages. None of the labourers interviewed have ever signed a work contract or joined a union, workers knew that they could be hired and fired at will. This organizational precarity means that most labourers have no room to stand up to their bosses and demand better conditions. To be sure, this is not unique to either of these livelihoods as this is a common pattern among various kinds of ‘informal’ work across Southeast Asia and contributes to precariousness for people and their livelihoods (Hewison and Kalleberg 2013).

**Table 3:** Understanding the livelihoods of sand labourers and coastal fishers through a precarity analysis

Livelihood type	Precarity components				
	Organizational context	Economic conditions	Level of labour protection	Temporal and spatial effects	Ecological and biophysical change
Sand labourers	No unions, workers can be fired at will, no work contracts signed	Higher pay than many rural resource jobs. (pumpers and dredgers earn $300 to $500 USD per month), but pay is unstable. Some workers are not paid in time	No protection against injury. In rare cases, financial protection from employers in times of low availability of sand work is offered	Work is uneven, starting and stopping without predictability. Many workers travel from station to station in search of work	Sand is still plentiful in Cambodia, but as it is depleted, workers will lose their jobs
Fishing	Most coastal fishers are self-employed. Fishers are not organized into unions, and have no formal work contracts	Fishers earn around $250–300 USD per month, varying highly between households and dependent on expenses incurred. Virtually all fishers have reported decreasing earnings over time	No protection against injury or equipment loss; no formal social protection measures. Insurance schemes are uncommon among rural fishing villages	As marine resources decline, fishers have to travel further out to sea, often spending the night or working over the night. Rainy season results in the halting of fishing activities for most	Fishers face declining catches over time. Sand mining, overfishing, and environmental change contribute to declining fish stocks

Both sand mining and fishing also follow a kind of ‘boom and bust’ cycle which, on the one hand, lends itself to adding an element of precarity, but on the other hand, acts as an allure with the potential of a big catch or payday driving the motivation for both groups of people. From an economic standpoint this plays out in the form of, on average, higher pay than for instance minimum wage work in Cambodia, but also lack of predictability in income. Although the sand industry offers an increased salary for most Cambodians coming from rural areas, workers still experience financial precarity and irregular pay. Another source of financial precarity comes from the frequent imbalances between sand supply and demand resulting in week- or month-long periods during which workers do not work nor receive any salary. All sand mining labourers interviewed mentioned that there were always periods of time, sometimes stretching months, where little sand was being delivered. Similarly, for fishers, in certain months, they can catch a lot of seafood but there are also several months where they largely cannot work. Unlike sand labourers that do not have to put capital upfront, the added economic dimension for fishers is that they must pay for all of their fishing-related expenses out of pocket and sometimes when these are subtracted from the value of their catch, they are left with meagre returns (Marschke 2012; Asif 2020). Related to organizational aspects, both forms of work offer little (if any) labour protections. If a fisher or sand labourer is injured, there is no scheme that can provide financial support and any length of time that they remain out of work represents lost income. The degree of precarity differs between the two as a fisher is arguably in a higher position of vulnerability compared to a sand labourer. For instance, if a fisher is out of work due to unforeseen mechanical issues, they cannot simply switch to another means of earning a living. By contrast, the sand labourer can try to find other work since they are primarily selling their labour. On the other hand, sand labourers are at the mercy of their employers for work and cannot simply choose to work longer hours if they want to earn more.

Temporal and spatial elements also play a central role. Sand work creates transient and relatively marginal livelihoods. Workers are often mobile, both geographically and in their occupations, but have few opportunities to attain higher-level jobs such as a sand barge captain. For sand dredgers particularly, sand work is often remote and cut off from society. Horizontal movement between less well-paid jobs is more frequent, with workers switching jobs and/or sand companies based on their preferences. For example, a sand pumper may decide to work in a more remote location as a sand dredger because this involves less physical labour, while a sand dredger may switch to being a sand pumper in order to live closer to or in Phnom Penh. In other instances, a captain may be ‘demoted’ to being a sand pumper in Phnom Penh because they are not willing to travel constantly up and down the river. For fishers, marine resource decline in areas where they would traditionally be able to catch enough fish has forced them to go farther distances out to sea, which has a two-pronged effect by exposing them to riskier outcomes if they are stranded in areas that see less frequent boat traffic and higher fuel costs, which can eat into their bottom line.

For decades, fishers have had to cope with a variety of ecological and environmental change, and so, for them, sand mining and its acute impacts on marine resources and thus their livelihoods, is not new, per say. If anything, it has been another factor in what was already a precarious livelihood. In some ways, what is happening in sand mining reflects the situation in coastal fisheries several decades ago where a seemingly boundless natural resource drove Cambodians to establish their livelihoods around it. While the demand for sand has fluctuated, it has driven an influx of labourers who now depend on it for their livelihood. However, over time, just as has been the case for fishers, the sand will eventually be depleted (or become increasingly costly to obtain), and eventually, sand labourers will be out of a job. The contradiction here is that sand mining is exacerbating precarity for fishers by causing acute, negative ecological change, while supporting the (ostensibly precarious) livelihoods of sand labourers. In other words, this represents a case where a natural resource exploitation has paradoxical impacts on livelihoods, supporting one group while directly undermining another. Yet, in doing so, it only exacerbates the long-term precarity of both fishers and sand labourers.

## Conclusion

Following Marschke et al. (2020), we add to the empirical insights to fill in the missing link within precarity analysis, specifically in the Global South, between ecological decline and precarity and how the former directly and indirectly contributes to precarious work in the case of coastal fishers. We bring in precarity as an analytical lens and descriptive tool, which gives people and their livelihoods a central focus and show the merit of this by using empirical insights from three coastal fishing villages and selected sights of sand mining along the Mekong. By connecting other livelihoods which are impacted by natural resource exploitation, and using a precarity analysis, we aim to make the case for expanding the focus of impacts by natural resource use beyond the activity, to the people and their livelihoods that are involved in the exploitation of these resources. Doing so reveals how an unconventional, and largely ‘invisible’ quest for a natural resource, namely the drive to extract sand from the Mekong River and coastal areas of Cambodia, is interwoven with fisheries and elucidates the interplay between precarious labour, resource extraction, and livelihoods. We hope this intersection motivates other researchers and scholars to look at other natural resource-based livelihood contexts in the Global South through the lens of precarity and how ecological decline can have impacts on livelihoods and working conditions beyond the resources themselves.

## Footnotes

1 In addition to the importance as a source of labour, the sector also provides the majority (50–80%) of the national dietary animal protein intake for Cambodians (Sensereivorth and Rady 2013; Estepa et al. 2016; Hap et al. 2016).

2 The majority of the sand mining in Cambodia since 2017 has been occurring in the Mekong River with sand mining in the coastal region having generally ceased since then.

3 This early exploitation for timber was largely confined to forests located at the proximity of accessible rivers which provided long-distance transport and eventual exportation (Le Billon 1999).

4 It is important to note that the exploitation of Cambodia’s forests largely escaped public taxation, fueling wealth disparities, inspiring corruption among the (poorly remunerated) civil servants, triggering local/regional conflict, and creating a host of environmental damage (biodiversity loss, erosion, etc.) (Le Billon 1999; McKenney and Tola 2002; Global Witness 2007).
